# Age-stratified association between serum uric acid and lumbar bone mineral density in elderly Chinese women with vertebral compression fractures: a cross-sectional analysis

**DOI:** 10.3389/fmed.2025.1591791

**Published:** 2025-09-05

**Authors:** Jianxiang Zhu, Zengbing Xia, Jikang Min, Wenlin Hu, Heng Li, Chao Mei

**Affiliations:** ^1^Department of Orthopedics, The First People's Hospital of Huzhou, Huzhou, China; ^2^Department of Endocrinology, The First People's Hospital of Huzhou, Huzhou, China

**Keywords:** serum uric acid, bone mineral density, vertebral compression fractures, elderly women, age stratification

## Abstract

**Objective:**

To investigate the association between serum uric acid (SUA) levels and lumbar bone mineral density (BMD) in elderly Chinese women with vertebral compression fractures (VCFs), with a specific focus on age-dependent variations in this association.

**Methods:**

We conducted a cross-sectional study of 490 female patients aged ≥60 years with VCFs. SUA levels served as the primary exposure variable, and lumbar spine BMD was the outcome variable. Multivariate linear regression models were employed to adjust for potential confounders, incorporating comprehensive subgroup analyses.

**Results:**

A statistically significant positive and independent correlation emerged between SUA levels and spine lumbar BMD after multivariable adjustment (*β* = 0.045, 95% CI 0.026–0.064). The association demonstrated pronounced variability across age groups, with a more robust correlation in patients younger than 75 years (*β* = 0.069, 95% CI 0.039–0.098) compared to those 75 years and older (*β* = 0.026, 95% CI 0.002–0.050), revealing a statistically significant interaction (*p* = 0.008).

**Conclusion:**

Our findings reveal a nuanced, age-dependent positive correlation between SUA levels and lumbar BMD in elderly Chinese women with vertebral compression fractures. These results suggest SUA may serve as a potential biomarker for fracture risk assessment and bone health evaluation, particularly in younger elderly populations.

## Introduction

Osteoporosis constitutes a significant global public health issue, with profound implications for elderly populations, particularly women. Epidemiological studies indicate a high prevalence of spinal and hip osteoporosis among Chinese women, with approximately 29.13% of individuals over the age of 50 experiencing notable reductions in bone density (1). In China, osteoporotic vertebral compression fractures (OVCFs) are identified as the most common type of fracture associated with osteoporosis, with an estimated annual incidence of 1,110,000 cases (2). These fractures significantly compromise patients’ quality of life, substantially increasing the risks of disability and mortality (3).

Contemporary research increasingly emphasizes the pivotal role of oxidative stress mechanisms in age-related bone metabolism deterioration (4, 5). Emerging evidence suggests that oxidative stress accelerates bone turnover through complex cellular mechanisms, specifically by impairing osteoblastogenesis, promoting disproportionate bone resorption, and disrupting bone mineral density maintenance (6–8). In postmenopausal women, oxidative stress emerges as a more critical determinant of bone loss compared to traditional hormonal deficiency models (9, 10). Serum uric acid (SUA), the terminal metabolite of purine metabolism, presents a nuanced antioxidant profile with context-dependent protective and potentially detrimental characteristics (11). Physiological SUA concentrations demonstrate potential anti-osteoporotic properties, suggesting a nuanced role in bone metabolism (12). Specifically, antioxidants like SUA may mitigate bone loss by modulating oxidative stress-induced cellular mechanisms, potentially preserving bone mineral density and structural integrity (8, 13).

Epidemiological investigations exploring the relationship between SUA and bone mineral density (BMD) have yielded conflicting and nuanced findings across diverse populations. Representative studies have demonstrated significant variations, including positive correlations in certain demographic groups, gender-specific associations, and contrasting findings that reveal no notable relationship between uric acid levels and bone density (14–16). Existing literature predominantly focuses on populations with preserved bone density, inadvertently overlooking individuals with compromised skeletal integrity. Vertebral compression fractures (VCFs) represent a significant morbidity in postmenopausal osteoporosis (14), with substantial implications for patient quality of life (15) and healthcare resource utilization (16, 17).

To address this critical knowledge gap, we conducted a comprehensive cross-sectional study of elderly Chinese women (aged ≥60 years) with vertebral compression fractures. Our primary objectives were to investigate the association between SUA and lumbar BMD, and explore the potential age-related variations in the SUA-BMD relationship.

## Materials and methods

### Study population

This cross-sectional study was designed to investigate the association between SUA levels and BMD among Chinese women aged 60 years and older with OVCFs.

Data were systematically and consecutively collected from a general hospital in southern China. Patient records were extracted from the electronic medical record system for the period between January 1, 2022, and December 31, 2024, adhering to predefined inclusion and exclusion criteria.

**Figure 1:**
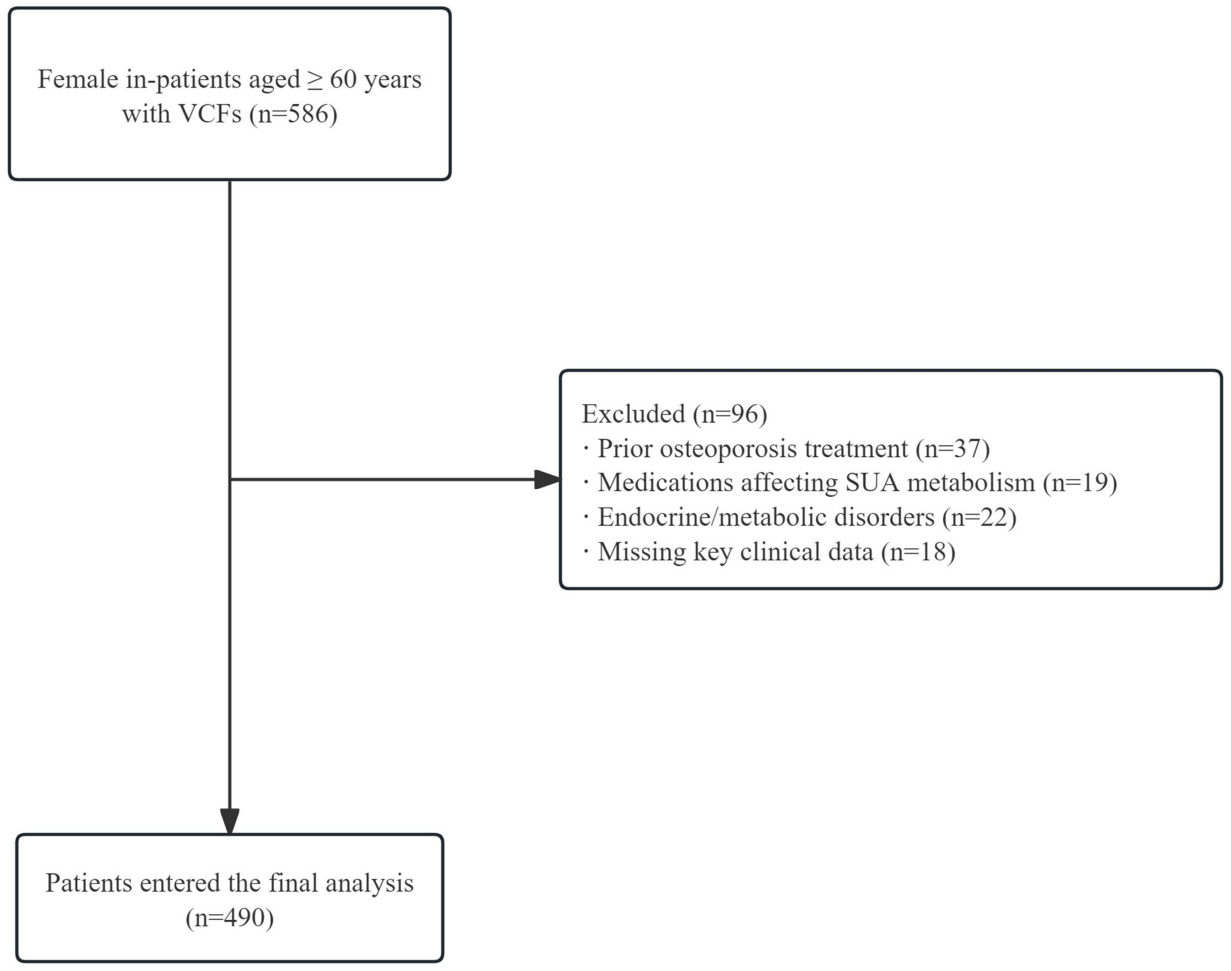
Participant selection flowchart. A total of 586 female patients aged ≥60 years with vertebral compression fractures were screened. After excluding 96 patients due to prior osteoporosis treatment, medications affecting SUA metabolism, endocrine/metabolic disorders, and missing clinical data, 490 patients were included in the analysis.

Inclusion criteria comprised: (1) female sex; (2) age ≥ 60 years; (3) confirmed acute OVCFs. Exclusion criteria encompassed: (a) prior osteoporosis treatment; (b) medications affecting uric acid metabolism; (c) endocrine or metabolic disorders; (d) lack of critical clinical data. The initial screening process comprehensively evaluated 586 potential participants. Following rigorous application of the predefined inclusion and exclusion criteria, a final cross-sectional sample of 490 participants was identified and analyzed. The complete participant flow is illustrated in Figure 1.

### Ethical considerations

The research protocol was meticulously reviewed and approved by the Institutional Review Board of the hospital (approval No.2025KYLL013-01). In alignment with the retrospective observational study design, the requirement for individual informed consent was waived, consistent with established ethical guidelines for minimal-risk retrospective medical record reviews.

All data collection and processing procedures were conducted with the utmost rigor, adhering to stringent patient privacy protection protocols. Personally identifiable information was comprehensively anonymized through a standardized de-identification process, ensuring complete confidentiality of patient data. Unique numerical identifiers were assigned to each participant, effectively decoupling individual patient information from the research dataset.

The study was executed in strict compliance with the Declaration of Helsinki’s ethical principles for medical research involving human subjects. Additionally, the research methodology was designed to minimize potential risks to participants and maintain the highest standards of data integrity and patient privacy.

### Laboratory measurements

Blood samples were collected from all subjects between 8:00 and 9:30 AM, following a fasting period of at least 8 h. Subsequent biochemical analysis of the blood samples was conducted using the ARCHITECT ci16200 Integrated System, assessing a range of parameters including: SUA, white blood cell (WBC), hemoglobin (HGB), red blood cell specific volume (HCT), blood platelets (PLT), alanine aminotransferase (ALT), aspartate aminotransferase (AST), glutamyl transpeptidase (GGT), total cholesterol (TC), triglycerides (TG), high-density lipoprotein (HDL), low-density lipoprotein (LDL), alkaline phosphatase (ALP), fasting blood glucose (FBG), albumin (Alb), C-reactive protein (CRP), serum creatinine (sCr), urea nitrogen (BUN), estimated glomerular filtration rate (eGFR), free triiodothyronine (FT3), free thyroxine (FT4), thyroid stimulating hormone (TSH), procollagen type I amino-terminal propeptide (PINP); collagen type 1 cross-linked C-telopeptide (*β*-CTx), osteocalcin (OC), 25-hydroxyvitamin D3 (25-OHD3), serum phosphorus (P), serum calcium (Ca), and intact parathyroid hormone (PTH). All collected blood samples were promptly sent to the hospital for analysis.

### Measurement of BMD

BMD of the lumbar spine was evaluated using dual-energy X-ray absorptiometry (DXA) on a Discovery-Wi system (S/N 88155) at the Bone Density Testing Laboratory of the hospital. All assessments were conducted by a single skilled operator utilizing the same equipment, following established protocols to minimize error. A daily quality control program was implemented prior to patient assessments, resulting in a coefficient of variation of 1.0% for lumbar spine measurements. The BMD measurements encompassed data from the first through fourth lumbar vertebrae (L1–L4), with values expressed in grams per square centimeter (g/cm^2^).

### Clinical evaluation and measurement

Vital signs were documented for each participant, and a comprehensive questionnaire was administered. The data recorded included age, height, weight, fracture site, cardiovascular events, cerebrovascular events, hypertension, diabetes, smoking status, alcohol consumption, educational level, and physical activity. Body mass index was computed as weight (kg) divided by the square of height (m^2^). They were evaluated at baseline, with assessments recorded within 24 h of hospital admission, immediately before any therapeutic interventions, including surgical, pharmacologic, or rehabilitative treatments.

### Statistical analysis

This study employed a comprehensive multi-stage statistical approach to evaluate the association between SUA and BMD. Statistical differences across SUA tertile groups were compared using χ2 test, One-Way ANOVA, and Kruskal-Wallis H test.

Regression analysis constructed three models: Model 1: without covariate adjustment; Model 2: adjusted for age and BMI; Model 3: fully adjusted (Table 1). Generalized Additive Model and smooth curve fitting (penalized spline method) were used to analyze the nonlinear relationship between SUA and BMD. When nonlinearity was detected, a recursive algorithm calculated the inflection point, and piecewise linear regression models were constructed, with the optimal model determined by log-likelihood ratio test.

**Table 1:** The association between SUA and lumbar BMD.

Exposure variable	Model 1 *β* (95% CI) *p* value	Model 2 *β* (95% CI) *p* value	Model 3 *β* (95% CI) *p* value
Serum Uric Acid	0.073 (0.055, 0.091) < 0.001	0.060 (0.043, 0.078) < 0.001	0.045 (0.025, 0.064) < 0.001
Serum Uric Acid Tertiles			
T1	Ref	Ref	Ref
T2	0.038 (0.003, 0.073) 0.032	0.029 (−0.004, 0.063) 0.090	0.015 (−0.020, 0.050)0.400
T3	0.149 (0.113, 0.184) < 0.001	0.122 (0.087, 0.157) < 0.001	0.090 (0.052, 0.129) < 0.001
P for trend	0.075 (0.057, 0.093) < 0.001	0.061 (0.043, 0.079) < 0.001	0.045 (0.026, 0.064) < 0.001

Model 1: Unadjusted.

Model 2: Adjusted for age and BMI.

Model 3: Fully adjusted for age, BMI, and multiple clinical covariates. *Covariates include: ALT, AST, TC, TG, HDL, AKP, FBG, CRP, 25-OHD3, P, Ca, PTH, fracture site, cardiovascular events, cerebrovascular events, hypertension, diabetes, smoking status, drinking status, education, and physical activity. Ref, Reference.

Subgroup analysis employed stratified linear regression models, converting continuous variables to categorical variables, performing interaction tests, and assessing effect modification using likelihood ratio test. Sensitivity analysis converted SUA to a categorical variable, calculated trend *p*-values to validate continuous variable analysis results and explore potential nonlinearity.

All the analyses were performed with the statistical software packages R (http://www.R-project.org, The R Foundation) and EmpowerStats (http://www.empowerstats.com, X&Y Solutions, Inc., Boston, MA). *p* values less than 0.05 (two-sided) were considered statistically significant.

## Results

### Baseline characteristics of selected participants

A total of 490 participants were included in the final data analysis after undergoing screening based on inclusion and exclusion criteria. The baseline characteristics of these participants, categorized according to tertile of SUA, are presented in Table 2. On average, the selected participants had an age of 75.76 ± 7.92 years. Statistically significant differences were observed in various factors, including BMI, cardiovascular events, hypertension, smoking status, drinking status, PLT, sCr, BUN, eGFR, HDL, Hcy, *β*-CTX, and lumbar BMD among different SUA groups (all *p* values < 0.05). Participants in the SUA (T3) group exhibited higher levels of age, BMI, WBC, HGB, HCT, GGT, ALB, P, Ca, sCr, BUN, TG, Hcy, BMD, as well as a greater prevalence of cardiovascular events, hypertension, diabetes, current smokers, and current drinkers compared to the other groups (Table 2).

**Table 2:** Baseline characteristics of participants.

Serum uric acid (mg/dL)	Total subjects *n* = 490	Q1 (1.20–2.60) *n* = 153	Q2 (2.70–3.30) *n* = 173	Q3 (3.40–6.10) *n* = 164	*p*-value
Demographic characteristics					
Age, years	75.76 (7.92)	75.93 (8.36)	75.36 (8.11)	76.01 (7.29)	0.713
Fracture site, *n* (%)					0.201
Thoracic vertebra	211 (43.06%)	74 (48.37%)	74 (42.77%)	63 (38.41%)	
Lumbar vertebra	279 (56.94%)	79 (51.63%)	99 (57.23%)	101 (61.59%)	
BMI, kg/m^2^	21.83 (3.60)	20.91 (3.24)	21.59 (3.44)	22.93 (3.80)	<0.001
Lifestyle characteristics					
Cardiovascular events, *n* (%)	76 (15.77%)	27 (18.12%)	17 (10.00%)	32 (19.63%)	0.035
Cerebrovascular events, *n* (%)	57 (11.83%)	22 (14.77%)	16 (9.41%)	19 (11.66%)	0.335
Hypertension, *n* (%)	252 (52.28%)	72 (48.32%)	82 (48.24%)	98 (60.12%)	0.048
Diabetes, *n* (%)	71 (14.73%)	23 (15.44%)	22 (12.94%)	26 (15.95%)	0.071
Smoking status, *n* (%)					0.004
Current	53 (10.88%)	9 (5.96%)	16 (9.30%)	28 (17.07%)	
Never	411 (84.39%)	136 (90.07%)	151 (87.79%)	124 (75.61%)	
Former	23 (4.72%)	6 (3.97%)	5 (2.91%)	12 (7.32%)	
Drinking status, *n* (%)					<0.001
Current	51 (10.49%)	6 (3.97%)	14 (8.19%)	31 (18.90%)	
Never	418 (86.01%)	137 (90.73%)	154 (90.06%)	127 (77.44%)	
Former	17 (3.50%)	8 (5.30%)	3 (1.75%)	6 (3.66%)	
Activity, *n* (%)					0.501
Active	244 (50.21%)	70 (46.36%)	88 (51.16%)	86 (52.76%)	
Sedentary	242 (49.79%)	81 (53.64%)	84 (48.84%)	77 (47.24%)	
Education, *n* (%)					0.412
Junior high school or lower	453 (93.98%)	142 (95.95%)	158 (92.40%)	153 (93.87%)	
Senior high school or higher	29 (6.02%)	6 (4.05%)	13 (7.60%)	10 (6.13%)	
Laboratory parameters					
WBC,10^9^/L	7.88 (2.73)	7.68 (2.70)	7.82 (2.72)	8.12 (2.76)	0.340
HGB, mg/L	122.75(17.07)	122.11 (15.74)	121.58 (16.88)	124.57 (18.36)	0.235
HCT, L/L	0.37 (0.05)	0.37 (0.05)	0.37 (0.05)	0.38 (0.05)	0.170
PLT, 10^9^/L	177.41 (59.33)	173.12 (62.35)	187.51 (59.96)	170.76 (54.52)	0.019
ALT, U/L	16.35 (9.36)	16.64 (9.65)	17.02 (10.88)	15.37 (7.02)	0.245
AST, U/L	20.78 (7.98)	20.98 (8.49)	20.86 (8.20)	20.51 (7.25)	0.858
GGT, U/L	25.41 (24.05)	24.92 (28.66)	24.98 (23.25)	26.32 (19.95)	0.839
ALP, U/L	89.03 (28.50)	90.39 (29.28)	90.67 (28.87)	86.04 (27.29)	0.257
Alb, g/L	38.74 (4.27)	38.59 (3.99)	38.66 (4.68)	38.98 (4.08)	0.686
FBG, mmol/L	6.69 (1.97)	6.76 (2.08)	6.68 (1.88)	6.63 (1.98)	0.842
P, mmol/L	1.04 (0.20)	1.03 (0.18)	1.03 (0.19)	1.05 (0.21)	0.552
Ca, mmol/L	2.28 (0.13)	2.27 (0.12)	2.27 (0.14)	2.29 (0.14)	0.277
sCr, mg/dL	0.80 (0.23)	0.72 (0.14)	0.76 (0.16)	0.91 (0.30)	<0.001
BUN, mmol/L	6.40 (2.06)	5.85 (1.65)	6.28 (1.74)	7.02 (2.50)	<0.001
eGFR, mL/min/1.73 m^2^	85.29 (20.18)	90.44 (19.68)	86.51 (17.18)	79.20 (22.02)	<0.001
TC, mmol/L	4.52 (0.97)	4.55 (0.93)	4.58 (0.99)	4.41 (0.99)	0.242
TG, mmol/L	1.24 (0.72)	1.19 (0.73)	1.25 (0.74)	1.27 (0.71)	0.649
HDL, mmol/L	1.35 (0.39)	1.44 (0.36)	1.34 (0.34)	1.29 (0.46)	0.003
LDL, mmol/L	2.56 (0.84)	2.51 (0.79)	2.65 (0.88)	2.51 (0.84)	0.213
CRP, mg/L	18.33 (24.91)	20.89 (27.70)	17.78 (25.25)	16.55 (21.58)	0.283
Hcy, umol/l	15.93 (8.70)	14.92 (7.34)	14.70 (8.10)	18.19 (10.01)	<0.001
FT3, pmol/L	3.98 (0.76)	4.01 (0.79)	3.97 (0.70)	3.96 (0.79)	0.814
FT4, pmol/L	13.83 (2.02)	13.92 (2.04)	13.94 (1.95)	13.62 (2.07)	0.333
TSH, pmol/l	1.77 (1.31)	1.87 (1.55)	1.67 (1.12)	1.79 (1.25)	0.436
PTH, pmol/l	67.85 (31.48)	67.58 (33.35)	68.45 (29.87)	67.47 (31.61)	0.953
β-CTx, ng/L	0.41 (0.24)	0.39 (0.21)	0.45 (0.28)	0.39 (0.22)	0.043
PINP, ng/L	60.63 (30.75)	57.92 (23.71)	64.39 (34.17)	59.14 (32.49)	0.152
OC, ng/L	14.33 (6.98)	14.12 (6.45)	14.81 (7.09)	13.99 (7.35)	0.553
25-OHD3, ng/L	21.31 (6.33)	20.39 (6.27)	22.06 (6.71)	21.36 (5.90)	0.078
Lumbar BMD, g/cm^2^	0.84 (0.17)	0.78 (0.16)	0.82 (0.15)	0.93 (0.18)	<0.001

Mean (SD) for continuous variables: the *p* value was calculated by the weighted linear regression model. (%) for categorical variables: the *p* value was calculated by the weighted chi-square test. IQR, interquartile range; BMI, body mass index; WBC, white blood cell; HGB, hemoglobin; HCT, red blood cell specific volume; PLT, blood platelet; ALT, alanine aminotransferase; AST, aspartate aminotransferase; GGT, glutamyl transpeptidase; ALP, alkaline phosphatase; Alb, albumin; FBG, fasting blood glucose; P, phosphorus; Hcy, Homocysteine; Ca, calcium; sCr, serum creatinine; BUN, urea nitrogen; eGFR, estimated glomerular filtration rate; TC, total cholesterol; TG, triglyceride; HDL, high-density lipoprotein; LDL, low-density lipoprotein; CRP, C-reactive protein; PINP, procollagen type I amino-terminal propeptide; PTH, intact parathyroid hormone; 25-OHD_3_, 25-dihydroxyvitaminD3; β-CTx, collagen type 1 cross-linked C-telopeptide; OC, osteocalcin; FT3, free triiodothyronine; FT4, free thyroxine; TSH, Thyroid Stimulating Hormone; BMD, bone mineral density.

### Association between SUA and BMD

We conducted a cross-sectional study to investigate the association between SUA and lumbar spine BMD. Our comprehensive statistical analysis revealed a significant positive correlation between SUA and BMD across multiple adjustment models (Table 1). In the unadjusted model (Model 1), each 1 mg/dL increment in SUA was associated with a 0.073 g/cm^2^ (95% CI: 0.055–0.091, *p* < 0.001) increase in lumbar BMD. After adjusting for age and BMI in Model 2, the association remained statistically significant, with a 0.060 g/cm^2^ (95% CI: 0.043–0.078, p < 0.001) increase per 1 mg/dL increment. In the fully adjusted model (Model 3), SUA remained an independent correlate of lumbar BMD: each 1 mg/dL increment in SUA was independently associated with a 0.045 g/cm^2^ (95% CI 0.025–0.064, *p* < 0.001) increase in lumbar BMD.

For the purpose of sensitivity analysis, we converted the SUA from continuous variable to categorical variable (Tertile of SUA), the P for trend of SUA with categorical variables in model 3 were consistent with the result when SUA is a continuous variable. We also found the trend of the effect size in different SUA groups was equidistant. To further confirm the linear relationship between SUA and BMD, we subsequently applied smooth curve fitting and the result of Generalized additive model showed that the relationship between SUA and BMD appeared linear after adjusting for confounding factors.

### Subgroup analysis

**Figure 2:**
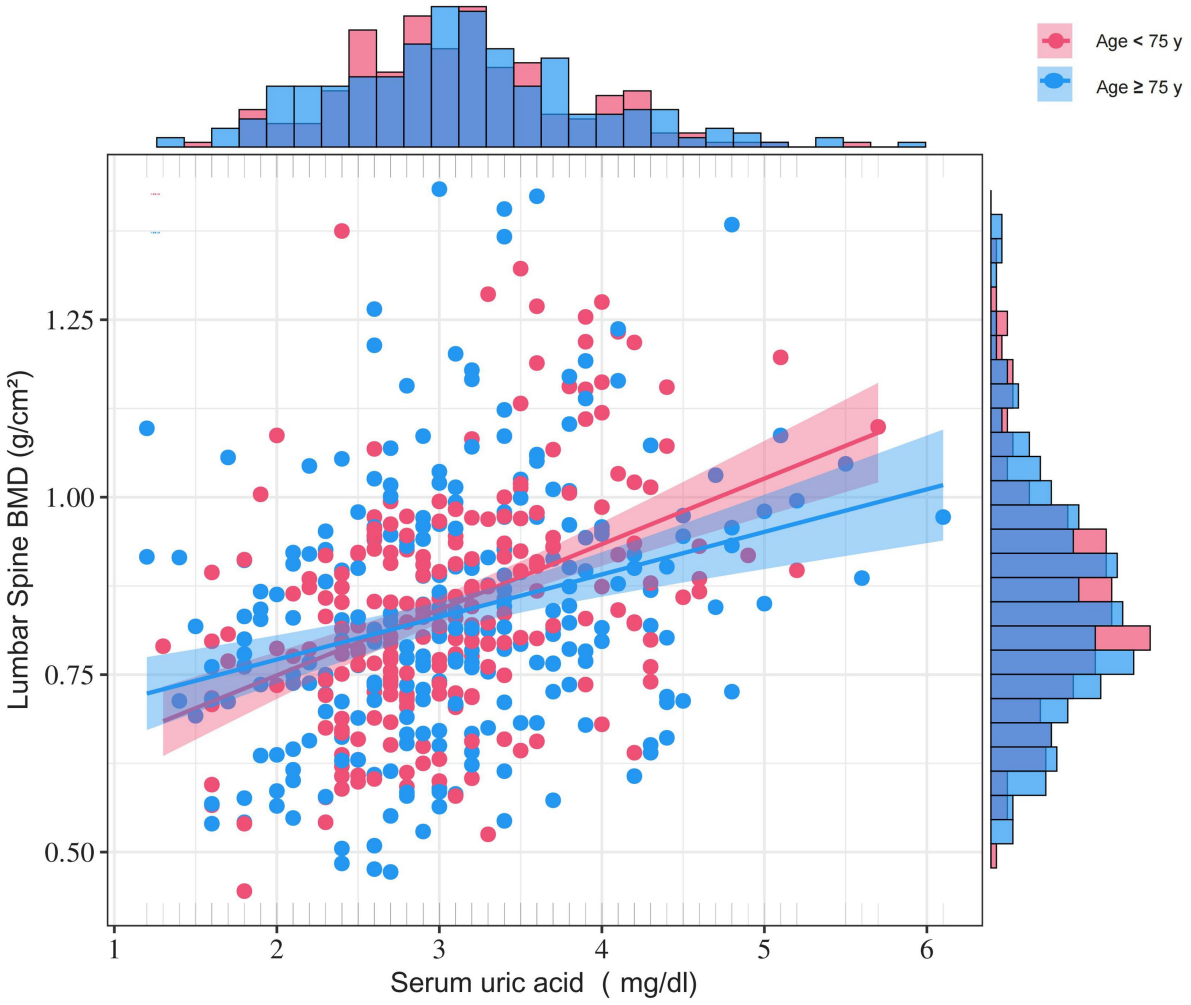
Age-stratified density-scatter plots illustrating the relationship between SUA and lumbar BMD. Each point represents an individual participant; the red and blue lines depict the fitted regression curves for women < 75 years and ≥ 75 years, respectively, with their corresponding red and blue shaded bands representing the 95% confidence intervals for each age group. Age, BMI, ALT, AST, TC, TG, HDL, AKP, FBG, CRP, 25-OHD3, P, Ca, PTH, fracture site, cardiovascular events, cerebrovascular events, hypertension, diabetes, smoking status, drinking status, education, and physical activity were adjusted.

**Figure 3:**
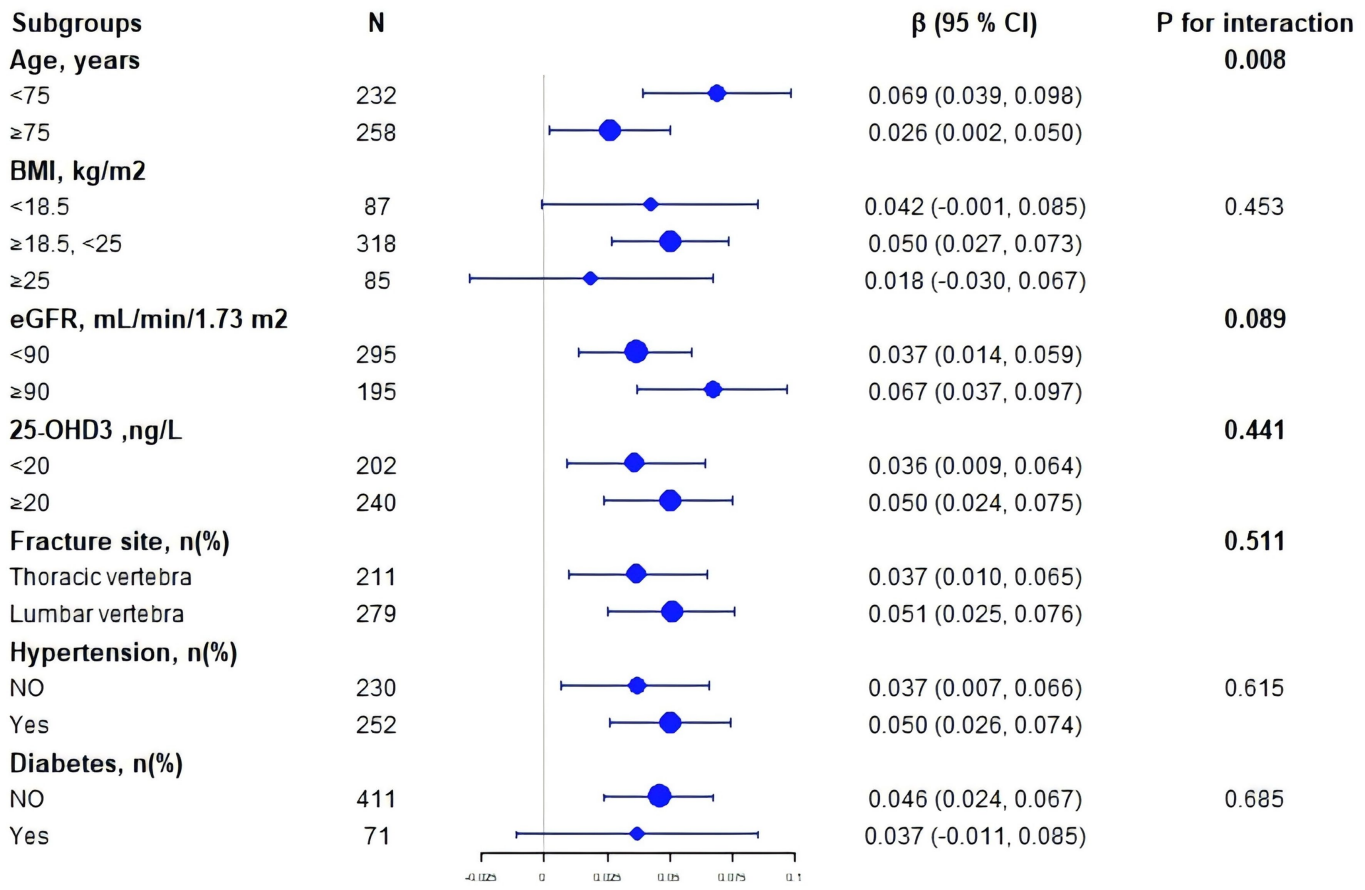
Subgroup forest plot of the SUA–lumbar BMD association. Each dot represents the *β*-coefficient (change in BMD per 1 mg/dL SUA increase); horizontal bars denote 95% CIs. The dashed vertical line shows the overall β from the fully adjusted model. P-interaction values indicate heterogeneity across strata.

We employed multiple stratification variables, including age, BMI, eGFR, 25-OHD3, fracture sites, hypertension, and diabetes, to explore potential effect size variations across these subgroups. In the age-stratified analysis, patients were categorized into two groups: those younger than 75 years and those 75 years or older. The 75-year cutoff was chosen based on epidemiological studies differentiating ‘young–old’ from ‘old–old’ individuals (18–21). The remaining stratification variables (BMI, eGFR, 25-OHD₃, fracture site, hypertension, and diabetes) were selected *a priori* based on published evidence indicating their independent associations with either SUA levels or BMD (22–24). While a significant association between SUA and BMD was observed in both age groups— < 75 years (*β* = 0.069, 95% CI 0.039–0.098) and ≥ 75 years (β = 0.026, 95% CI 0.002–0.050)—a statistically significant interaction was detected (P for interaction = 0.008) (Figure 2). Notably, subsequent subgroup analysis across BMI, eGFR, 25-OHD3 levels, fracture sites, hypertension, and diabetes revealed no significant interactions (P for interaction > 0.05), suggesting that age may be the primary modifier of the SUA-BMD relationship (Figure 3).

## Discussion

To our knowledge, this is the first study to delineate an age-differential association between SUA and lumbar BMD exclusively in postmenopausal women with acute OVCFs. We observed a robust, linear, independent, and positive SUA-BMD relationship that was markedly attenuated beyond 75 years.

SUA is the terminal metabolite of purine catabolism and accounts for up to 60% of plasma antioxidant capacity under physiological conditions (25). Oxidative stress is a well-established driver of osteoblast apoptosis and osteoclast activation (4, 26). In osteoporotic bone, where reactive oxygen species accumulate, SUA can neutralise peroxynitrite and hydroxyl radicals, thereby preserving osteoblast mitochondrial function and mineral apposition rate (5). Following vertebral fracture, the local bone environment becomes highly catabolic: osteoclastic resorption accelerates and bone turnover surges (27). Within this activated milieu, uric acid—an endogenous antioxidant—exerts amplified regulatory effects on osteoblast and osteoclast function, thereby magnifying its impact on bone metabolism (28). The steeper regression slope observed in women < 75 years supports the concept that residual antioxidant reserves are still sufficient for SUA to exert measurable skeletal protection, whereas age-related depletion of global antioxidant capacity attenuates this effect in the “old–old” subset (29, 30).

Consistent with recent studies in community-dwelling elderly women without osteoporosis or prior fracture (31, 32), we confirm a positive SUA–BMD association; however, effect sizes in these reports remain relatively small. By restricting our analysis specifically to osteoporotic fracture female patients, we significantly amplified the signal-to-noise ratio and revealed a strikingly larger effect (*β* = 0.045 g/cm^2^). As mentioned above, this amplification is biologically reasonable. The significant age interaction we observed further resolves prior inconsistencies: studies recruiting subjects >75 years often report no significant association (33), whereas those including “young–old” participants consistently demonstrate robust associations (34, 35). However, beyond demographic characteristics, the conflicting findings of these studies are likely attributable to differences in research design, population samples, controlled confounding factors, and potential methodological limitations. Consequently, while SUA appears beneficial across the entire spectrum of postmenopausal bone health, its quantitative contribution to fracture-risk stratification is likely contextual—higher in the high-turnover, post-fracture state than in low-turnover, non-osteoporotic elderly women. Prospective cohorts that directly compare these two strata are required to confirm the differential effect size and to determine whether SUA offers incremental predictive value in the absence of osteoporosis.

Compared with established biomarkers for the early assessment of osteoporotic risk, SUA is inexpensive and universally available. The International Osteoporosis Foundation endorses serum procollagen type-I N-terminal pro-peptide (PINP) and C-terminal telopeptide of type-I collagen (*β*-CTX) as indices of bone formation and resorption, yet these assays require dedicated platforms and are susceptible to circadian variability (36). Antioxidant nutrients such as 25-hydroxyvitamin D and vitamin C correlate positively with bone mineral density (37, 38), but their serum concentrations fluctuate with season and supplementation (39). Maggio et al. (26) demonstrated a marked reduction in total plasma antioxidant capacity in elderly women with osteoporosis, implying that antioxidant micronutrients could confer skeletal protection; however, individual antioxidant parameters showed inconsistent associations with fracture risk. SUA, an endogenous antioxidant, is quantified concurrently in routine biochemistry panels without additional cost. Notably, the antioxidant efficacy of SUA may diminish with advancing age, and it remains unclear whether SUA offers incremental predictive value beyond PINP, β-CTX, or 25-hydroxyvitamin D. Longitudinal cohorts or mechanistic intervention studies are therefore required to establish the independent and additive utility of SUA for personalised fracture-risk assessment.

Our cross-sectional study demonstrates robust methodological strengths through a rigorous design and advanced statistical analysis. To address inherent limitations, we enrolled 490 elderly women with OVCFs, ensuring a representative sample. We used advanced statistical methods, including multivariate linear regression and generalized additive models, to minimize biases typical of cross-sectional studies. Our age-stratified analysis revealed important age-dependent patterns in the SUA and BMD relationship, supported by a significant interaction test (*P* interaction = 0.008). By controlling for over 20 clinical variables, we adopted a careful approach that provides strong scientific evidence despite the cross-sectional design limitations. While our study cannot establish causation, it offers a crucial foundation for future prospective research.

Our research has several methodological limitations that require cautious interpretation. First, as a single-centre study restricted to postmenopausal women with acute OVCFs, our findings have limited generalisability. Because data from fracture-free elderly women are absent, we cannot determine whether the observed SUA–BMD association extends to non-osteoporotic populations. Future case–control studies that compare SUA levels and BMD in women with and without fractures are therefore required to clarify the broader applicability and incremental value of SUA in elderly cohorts. Second, due to the cross-sectional design, we cannot establish a causal link between SUA and BMD. Third, while we adjusted for various potential confounders, unmeasured factors may still affect the results. Additionally, the study focuses on elderly Chinese women, which limits the applicability of our findings to other age, racial, or gender groups. Therefore, our results should be seen as exploratory and specific to postmenopausal women with OVCFs, potentially limiting generalizability to those without fractures. Future research should include fracture-free cohorts to assess the broader relevance of SUA as a biomarker for bone health.

## Conclusion

Our systematic analysis revealed the complex age-dependent association between SUA and BMD among elderly women with OVCFs. This finding not only enriches our understanding of the mechanisms of bone metabolism but also provides new biomarker references for early risk assessment and personalized interventions, especially in patients under 75 years of age. Despite the inherent limitations of a cross-sectional study design, our research lays a crucial foundation for future in-depth investigations. We cautiously recommend considering SUA as a potential risk indicator or auxiliary biomarker for bone density assessment, especially in younger elderly female populations. Future research should design prospective cohort studies to explore the precise mechanisms of SUA in bone metabolism regulation and develop personalized risk prediction models based on biological markers.

## Data Availability

The raw data supporting the conclusions of this article will be made available by the authors, without undue reservation.
