# Supplementary figures and images for: Age-stratified association between serum uric acid and lumbar bone mineral density in elderly Chinese women with vertebral compression fractures: a cross-sectional analysis
Source: Front Med (Lausanne). 2025 Sep 5;12:1591791. doi: 10.3389/fmed.2025.1591791 (PMC12446221; doi:10.3389/fmed.2025.1591791)

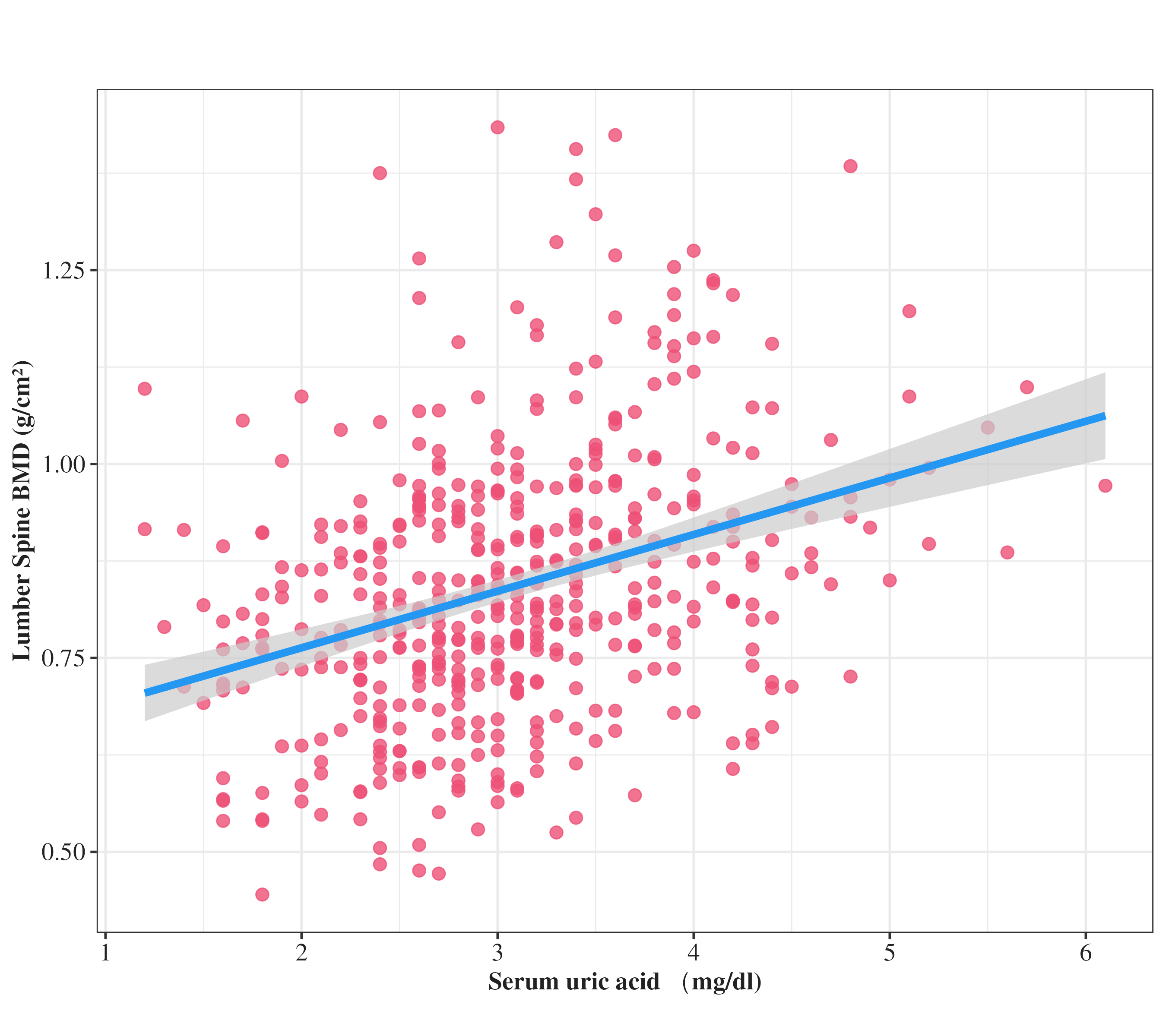

Supplement: Supplementary file 1 [file Image_1.TIFF]
