# Supplementary material for: Age-stratified association between serum uric acid and lumbar bone mineral density in elderly Chinese women with vertebral compression fractures: a cross-sectional analysis
Source: Front Med (Lausanne). 2025 Sep 5;12:1591791. doi: 10.3389/fmed.2025.1591791 (PMC12446221; doi:10.3389/fmed.2025.1591791)
Supplement: Supplementary file 2 [file Table_1.DOC]

Supplementary Figure S1. The association between SUA and lumbar BMD. Individual observations are depicted as red dots; the solid blue line represents the fitted curve, and the grey shaded area indicates the 95 % confidence interval. Age, BMI, ALT, AST, TC, TG, HDL, AKP, FBG, CRP, 25-OHD3, P, Ca, PTH, fracture site, cardiovascular events, cerebrovascular events, hypertension, diabetes, smoking status, drinking status, education, and physical activity were adjusted.
